# Large Traumatic Pneumatocele in a 2-Year-Old Child

**DOI:** 10.1155/2013/940189

**Published:** 2013-09-25

**Authors:** N. K. Cheung, A. James, R. Kumar

**Affiliations:** ^1^Department of Surgery, John Hunter Hospital, New Lambton Heights, Newcastle, NSW 2305, Australia; ^2^Department of Cardiothoracic Surgery, John Hunter Hospital, New Lambton Heights, Newcastle, NSW 2305, Australia; ^3^Department of Paediatric Surgery, John Hunter Children's Hospital, New Lambton Heights, Newcastle, NSW 2305, Australia

## Abstract

Traumatic pneumatoceles are a rare complication of blunt chest trauma in children. Although they characteristically present as small, regular shaped lesions which can be safely treated nonoperatively, larger traumatic pneumatoceles pose diagnostic and management difficulties for clinicians. This case study reports one of the largest traumatic pneumatoceles reported to date in the paediatric population, which resulted in aggressive surgical intervention for both diagnostic and treatment reasons. This case adds further evidence to the current literature that significantly large traumatic pneumatoceles with failure of initial conservative management warrant surgical exploration and management to optimise recovery and prevent complications.

## 1. Introduction

Traumatic pneumatocele (TP) is a rare condition occurring after blunt chest trauma in children and young adults, accounting for 3.9% of paediatric blunt chest traumas. In the literature, it has been described as traumatic pneumatoceles, traumatic lung cysts, pulmonary cavitations, cavitating haematoma, and traumatic pulmonary pseudocysts [1–6]. TP is characterized by the appearance of pulmonary cavities with no epithelial lining filled with air, fluid or, blood seen on radiology imaging, which usually resolve without surgery. It is commonly associated with pulmonary contusions but represents more extensive tissue disruptions and severity of injuries than a simple contusion [2, 3]. Clinical presentations, often seen within the first three to seven days after injury, include chest pain, cough, haemoptysis and dyspnea, and rarely irritability and mental changes [2, 3]. Conservative treatment is recommended when TP can be correctly diagnosed [2, 7], although dilemmas with their optimal management can arise with more complicated cases due to a paucity of paediatric case studies described in the literature. This report presents an unusual case of a very large traumatic pneumatocele resulting in surgical management to improve recovery time and to exclude serious underlying pathology and complications.

## 2. Case Presentation

**Figure 1:**
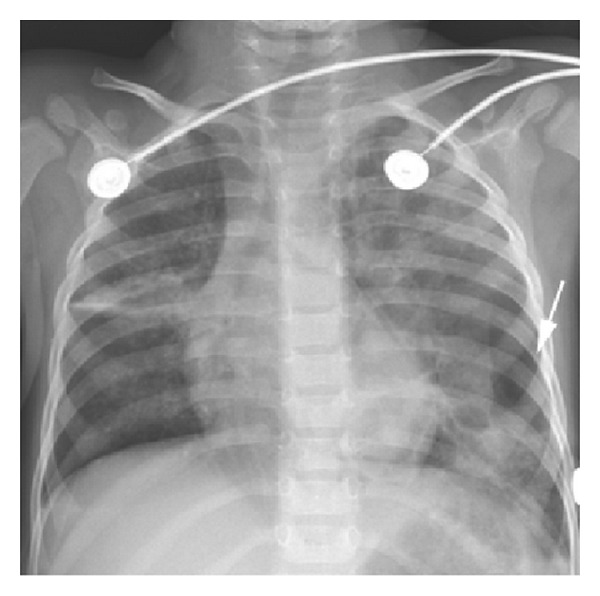
Chest X-ray showing large radiolucent and patchy cystic infiltration on the left lung field.

**Figure 2:**
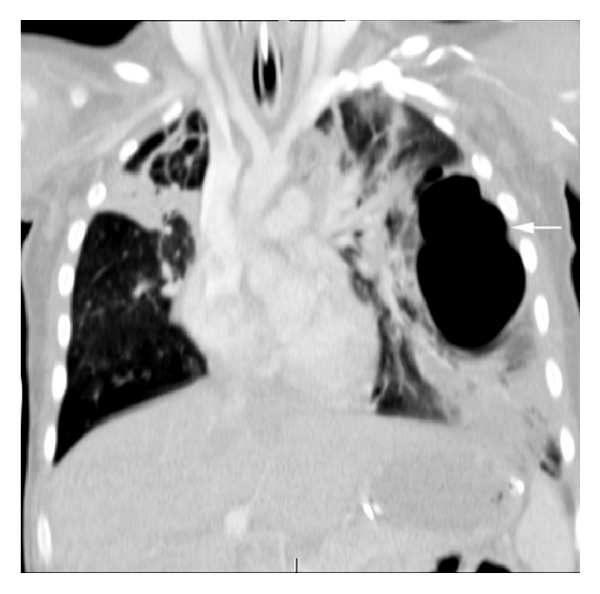
Coronal view on CT chest: a large and irregular pneumatocele extending throughout the left lung with extensive pulmonary contusions.

**Figure 3:**
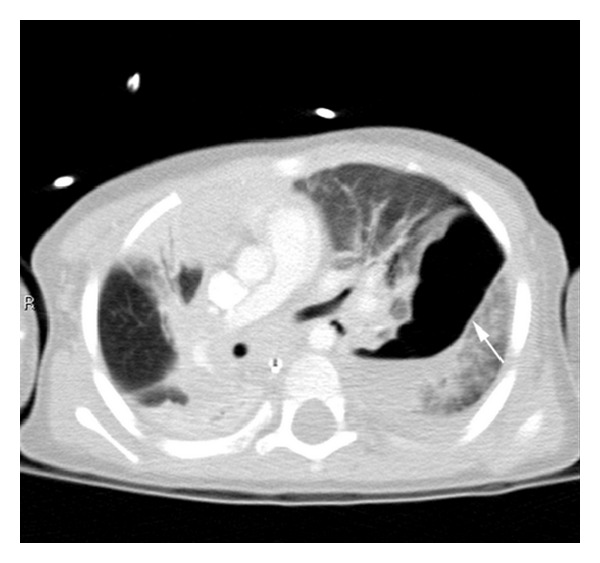
Axial view on CT chest. The pneumatocele measured approximately 6 cm (height) × 3 cm (width) × 4 cm (anteroposterior dimension).

A 2-year-old boy presented to the emergency department after being knocked over and his left shoulder and chest trapped under the rear wheels of a reversing car. He was shocked on arrival and had bruising on the left shoulder and chest, along with widespread petechiae across his face and eyes. Chest X-ray after resuscitation revealed bilateral contusions (Figure 1). A chest tube was placed for a suspected left pneumothorax. CT scan later demonstrated a massive and irregular traumatic pneumatocele extending throughout the left lung with extensive bilateral pulmonary contusions (Figures 2 and 3).

The child's clinical and respiratory status rapidly deteriorated, with maximum flow oxygen barely maintaining his oxygen saturations over 90%. He was intubated and ventilated; despite intensive support and conservative management over 24 hours, his clinical progress worsened. There was no radiographic improvement demonstrated, with concerns of a large haematocele or haemopneumothorax complicating the pneumatocele due to falling haemoglobin levels. There was additionally a concern of associated severe injuries, such as a ruptured hemidiaphragm, which could not be ruled out. Based on these findings, an urgent left thoracotomy was performed for exploration and for surgical repair.

At surgery, a large pneumatocele with a complicating haematocele (blood filled cavity) was found in the left lower lobe of the lung. The pneumatocele was opened, haematocele drained, and the cavity repaired with sutures. Postoperative period was uneventful, and the child was discharged on the seventh day. Six months after injury, the child had no residual lung parenchymal injury.

## 3. Discussion

Blunt chest trauma is a serious injury in the paediatric population presenting in a large spectrum of associated injuries ranging from lung contusions, pneumothorax, and oesophageal and cardiac injuries or more seriously, diaphragmatic and aortic ruptures [1, 2]. TP is generally classified as a benign condition representing more extensive tissue disruptions and injury severity than pulmonary contusions and is characterized by pulmonary cavities with no epithelial lining filled with air, fluid, or blood seen on plain X-ray or CT [2, 3]. 

The aetiology of TP is considered to be a two-step mechanism. (1) Blunt chest trauma results in rapid compression of a lung area, leading to barotraumas and ruptured parenchyma. (2) Elastic recoil of the lungs results in increased negative intrathoracic pressure leading to laceration formation. Localised increase in pressure in these lacerated areas causes surrounding lung parenchyma to retract. Formed cavities filled with fluid, blood, or air continue to increase in size until a balance of lung pressures is achieved between the cavity and surrounding tissue. In children and young adults, high chest wall compliance leads to increased transmission of forces and increased severity of injury [4, 5]. 

TP can present asymptomatically or with nonspecific symptoms including haemoptysis, chest pain, dyspnoea, cough, mild fever, and leukocytosis usually occurring 12 to 36 hours after trauma [2, 3, 7]. Diagnosis on a chest X-ray has low sensitivities of 24%. CT is much more accurate with a reported sensitivity of 96% [6]. This is consistent with the findings of this case study; the child's X-rays did not clearly identify a large TP possibly due to masking from pulmonary contusions.

Our review of the English literature identified 80 reported cases of TP since 1940. TPs reported in the literature are generally small, regular shaped lesions mostly treated conservatively [8]. Clinical observation is largely successful with few reported complications such as infections, secondary haematoceles, respiratory deterioration, size increase, or failure to resolve [3].

The TP in this case study measured 6 cm (height), 3 cm (width), and 4 cm (anteroposterior) in size. This is one of the largest cases reported in the literature, occupying over a third of this child's left chest. This atypical TP posed diagnostic dilemmas. It has previously been reported that complications can arise from large TP greater than 4 cm in diameter, including infections, haematocele development, respiratory compromise and deterioration, increased rates of mechanical ventilation insufficiencies, and failure to resolve with conservative management [3–5, 7, 9, 10]. Given the child's failure of initial conservative management and the concern of associated injuries from blunt chest trauma including traumatic diaphragmatic ruptures and oesophageal injuries with pneumomediastinum [8], explorative surgery was decided upon. Although this child's CT did not seem to indicate a diaphragmatic rupture, it has been reported however, that diaphragmatic ruptures are notoriously difficult to diagnose with only 53% seen on X-rays and wide ranges of CT sensitivities and specificities ranging from 54 to 73% [11, 12]. 

The operative repair of this child's TP likely significantly improved postinjury recovery time. The child was discharged uneventfully at day 7 after his operation. Surgery appeared to be of benefit. Intraoperatively, the pneumatocele seen on CT was complicated by a large haematocele, which was drained and surgically repaired. Surgery for complicated pneumatoceles is supported in the literature. Chon et al. described the mean resolution time for uncomplicated TP as 25.3 days, and mean resolution time for complicated blood filled pneumatoceles as 145.8 days [3]. Large TPs measuring greater than 4 cm in diameter without surgical management have also been reported to persist as a large cavity, resulting in respiratory insufficiencies, increased infection risks, and significantly longer resolution time [3, 7]. It is likely that large TPs have greater incidence of complications of haematoceles, such as the one found in this case study. Persisting cavities have been reported for large TPs managed non-operatively, whilst some warranted late thoracotomies at 6 months to correct this abnormality [13]. This further supports the initial surgical management of large TPs.

TPs are rare and relatively benign conditions that may develop following blunt chest trauma. Although diagnosis of TPs is in most cases straightforward, they continue to pose a dilemma to clinicians in more complex cases. Whilst evidence for the treatment of TPs exists solely as case series and reports in the literature, current evidence favours surgical management of particularly large and complex cases in order to prevent potential complications. This current case similarly emphasizes that large traumatic pneumatoceles potentially warrant surgical exploration and repair if early conservative management fails. Suspicion of associated injuries must always be considered with blunt chest traumas, whilst large TPs greater than 4 cm in diameter appear to be sufficient grounds to warrant consideration of surgical management.
